# A noninvasive and comprehensive method for continuous assessment of cerebral blood flow pulsation based on magnetic induction phase shift

**DOI:** 10.7717/peerj.13002

**Published:** 2022-02-23

**Authors:** Lingxi Zeng, Gen Li, Maoting Zhang, Rui Zhu, Jingbo Chen, Mingyan Li, Shengtong Yin, Zelin Bai, Wei Zhuang, Jian Sun

**Affiliations:** 1 School of Pharmacy and Bioengineering, Chongqing University of Technology, Chongqing, China; 2 College of Biomedical Engineering, Army Medical University, Chongqing, China; 3 College of Artificial Intelligence, Chongqing University of Technology, Chongqing, China

**Keywords:** Strokes, Noninvasive continuous assessment, Cerebral blood flow, Magnetic induction phase shift

## Abstract

Cerebral blood flow (CBF) monitoring is of great significance for treating and preventing strokes. However, there has not been a fully accepted method targeting continuous assessment in clinical practice. In this work, we built a noninvasive continuous assessment system for cerebral blood flow pulsation (CBFP) that is based on magnetic induction phase shift (MIPS) technology and designed a physical model of the middle cerebral artery (MCA). Physical experiments were carried out through different simulations of CBF states. Four healthy volunteers were enrolled to perform the MIPS and ECG synchronously monitoring trials. Then, the components of MIPS related to the blood supply level and CBFP were investigated by signal analysis in time and frequency domain, wavelet decomposition and band-pass filtering. The results show that the time-domain baseline of MIPS increases with blood supply level. A pulse signal was identified in the spectrum (0.2–2 Hz in 200–2,000 ml/h groups, respectively) of MIPS when the simulated blood flow rate was not zero. The pulsation frequency with different simulated blood flow rates is the same as the squeezing frequency of the feeding pump. Similar to pulse waves, the MIPS signals on four healthy volunteers all had periodic change trends with obvious peaks and valleys. Its frequency is close to that of the ECG signal and there is a certain time delay between them. These results indicate that the CBFP component can effectively be extracted from MIPS, through which different blood supply levels can be distinguished. This method has the potential to become a new solution for non-invasive and comprehensive monitoring of CBFP.

## Introduction

Stroke has become a global public health threat. In recent years, the incidence of hemorrhagic stroke has gradually decreased, while ischemic stroke has shown explosive growth (Phipps & Cronin, 2020). In 2018, the American Heart Association/American Stroke Association pointed out that maintaining the normal blood flow supply level of the cerebral artery is the key to the treatment of ischemic stroke (Powers et al., 2018). Therefore, real-time continuous monitoring of the cerebral blood flow (CBF) is of great importance, which provides guidance for suitable therapy and improving prognosis.

At present, there is no safe and acceptable method for continuous monitoring of the CBF in real time. Clinically, CT, MRI and other imaging methods are commonly used to evaluate the blood flow and infarction. However, traditional imaging equipment is bulky and cannot perform continuously monitoring tasks. Commonly, patients are arranged for imaging examination by doctor’s judgment or a pre-defined standardized schedule, which may easily lead to treatment delay (Ehntholt et al., 2020). Intracranial pressure (ICP) monitoring can indirectly reflect changes in CBF through brain volume. However, after stroke attack, the intracranial compensation mechanism can keep ICP within a certain range for a long period (Li et al., 2020; Ren et al., 2020). Only when the occlusion becomes severe and causes a large area of infarction, intracranial hypertension occurs rapidly. The dual-source Doppler volumetric ultrasound can be operated manually, but it is difficult to monitor for a long time. The transcranial Doppler (TCD) focuses on the cerebral blood flow velocity (CBFV) in the cerebral vessels which is considered to be an estimated value for CBF evaluation in the middle arteries and is often used for intermittent monitoring (Castro et al., 2017; Rasulo et al., 2017). However, when sympathetic nerve stimulation or infusion of vasoactive drugs triggers variation in the diameter of the measured middle cerebral artery, it is difficult to get accurate TCD results. The cortical laser Doppler flowmeter can also measure the blood flow rate in blood vessels. But the detection depth is limited and the brain tissue needs to be exposed (Rodriguez et al., 2018). Besides, the results are vulnerable to environmental factors. Near-infrared Spectroscopy (NIRS) can continuously and noninvasively monitor CBF by measuring the changes of blood oxygen and deoxyhemoglobin in blood vessels (Mathieu et al., 2020; Yang et al., 2019). The prerequisite is that the amount of light scattering remains unchanged and changes in attenuation are only caused by absorption. However, the composition of the intracranial tissue after ischemic stroke will change significantly, resulting in changes in both light absorption and scattering.

Magnetic induction phase shift (MIPS) is an emerging method in recent years that applies a low-frequency primary magnetic field on bio tissues to generate the induced current and form the secondary magnetic field (Mehrotra, Chatterjee & Sen, 2019). There is a phase shift between the primary field and the secondary field, namely MIPS, which is related to the electrical conductivity changes of bio tissues. Driven by normal arterial blood pressure, intracranial blood flow volume will produce a certain range of periodic pulsation with the heartbeat. In a cardiac cycle, the conductivity caused by the change of CBF volume is more significant than that of other intracranial components. Therefore, MIPS method is expected to monitor this cardiogenic cerebral blood flow pulsation (CBFP) signal. It is of great significance to guide personalized blood pressure management and treatment plans for patients with ischemic stroke by this method. Chen, Yan & Qin (2017) and Chen et al. (2017) parallelly conducted MIPS monitoring and MRI imaging experiments on rabbits and found that MIPS signals can quantitatively identify the main components of the brain such as cerebrospinal fluid (CSF) and CBF at different stages of cerebral hemorrhage. Griffith et al. (2018) developed a passive skin patch sensor with self-resonant frequency response. The physical model and volunteer experiments showed that it can non-invasively monitor brain volume changes. Oziel, Korenstein & Rubinsky (2018) analyzed the differences between a single-coil configuration and a planar helical antenna configuration in detecting the changes of intracranial hemorrhage volume. The results showed that the helical structure was suitable for the measurement of known bleeding points on the centerline, and the single-coil structure is suitable for the measurement of unknown bleeding points (Oziel, Korenstein & Rubinsky, 2018). Xu et al. (2019) built a portable MIPS detection system based on multi-channel antenna. They also proved the feasibility of MIPS in distinguishing hemorrhagic stroke and ischemic stroke through animal experiments. These researches provided strong evidence that MIPS has potential in diagnosis and monitoring of brain diseases. However, few studies have focused on the extraction of CBFP signal. The qualitative relationship between CBFP and MIPS is unknown.

In this study, we combined the theory of haemodynamics and the principle of MIPS detection to develop a new solution for non-invasive, continuous and comprehensive assessment of CBFP. The monitoring experiments of CBFP at different levels were carried out based on a self-made physical model of middle cerebral artery (MCA) and MIPS measurement system. Furthermore, we conducted MIPS and ECG synchronous monitoring trials on healthy volunteers. After signal processing including spectrum analysis, wavelet decomposition and band-pass filtering, we investigated the qualitative relationship between the parameters related to CBFP and the characteristics of MIPS signal in time and frequency domains.

## Materials and Methods

### Measurement principle

**Figure 1:**
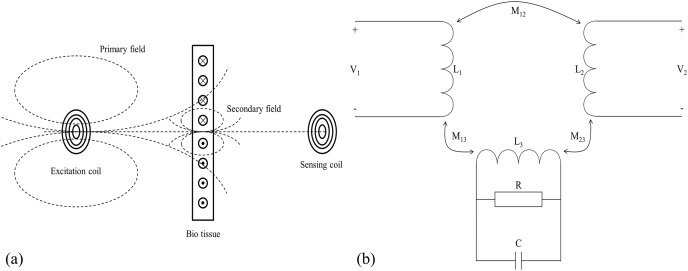
Measurement principle of magnetic induction phase shift. (A) Schematic diagram of MIPS. (B) Equivalent circuit diagram.

The schematic diagram of the magnetic induction phase shift (MIPS) method is shown in Fig. 1. According to Griffiths (2001) and Griffiths, Stewart & Gough (1999), the skin depth of the measured object in the electromagnetic field is expressed by the Eq. (1).

(1) $$\delta = \sqrt {{2 \over {\omega {\mu _0}\sigma }}}$$

where $\delta$ is the skin depth (mm), $\omega$ is the angular frequency of the excitation signal, ${\mu _0}$ is the magnetic permeability (H/m), and $\sigma$ is the electrical conductivity (S/m). When there is an excitation coil in free space with sinusoidal wave excitation ${V_1}$, the coil generates an alternating primary magnetic field in its surrounding space, denoted as $B$. If there is bio tissue in its vicinity, the tissue can be treated as the dielectric. The primary magnetic field $B$ induces eddy currents in bio tissue, which in turns generates a secondary magnetic field, denoted as $\Delta B$. If the geometry of the measured object is much smaller than the skin depth, the main magnetic field is approximated as a quasi-static field. At the position opposite to the excitation coil, the proportional relationship between the secondary magnetic field and the primary magnetic field is as follows:

(2) $${{\Delta B} \over B} = P\omega {\mu _0}\left[ {\omega {\epsilon _0}{\epsilon _r} - j\sigma } \right] + Q({\mu _r} - 1)$$

where ${\epsilon _0}$ is the dielectric constant in vacuum, ${\epsilon _r}$ and ${\mu _r}$ are respectively the relative permittivity and the relative permeability of bio tissue, $P$ and $Q$ are the constants related to the geometry of bio tissue. Consequently, when there is a receiving coil on the opposite side of the measured object, it will receive both $B$ and $\Delta B$, and respectively induces voltages $V$ and $\Delta V$ ( ${V_2} = V + \Delta V$). According to Eq. (2), the ${\epsilon _r}$ and $\sigma$ affect the real and imaginary parts respectively. Since biological tissue can only generate a very weak induced current under the action of $B$, the $\Delta B$ is also very weak, that is, $\Delta B$ ≪ $B$. The real part changes much less than the imaginary part (Griffiths et al., 2007). There is a relationship between induced voltage and magnetic field:

(3) $${{\Delta V} \over V} = {{\Delta B} \over B} = P\omega {\mu _0}\sigma$$

The $\sigma$ of biological tissues is much lower than that of good conductors such as metals, so the secondary magnetic field is indeed very weak. It is very difficult to directly measure the intensity change of the secondary magnetic field. Therefore, we focus on measuring the phase shift $\Delta \varphi$ between the superimposed magnetic field received by the receiving coil and $B$. That is the MIPS signal. The relationship between $\Delta \varphi$ and $\sigma$ is shown in Eq. (4).

(4) $$\varphi = \left|{{\Delta B} \over B}\right| \propto P\omega \sigma$$

The $\Delta \varphi$ is proportional to conductivity and excitation signal frequency. When the excitation signal frequency is constant, $\Delta \varphi$ reflects the conductivity of the measured tissue. The blood periodically pumped by the heart enters the brain through the carotid and vertebral arteries driven by vascular pressure, forming a dynamic CBFP circulation. During this circulation, CBFP changes periodically with the same frequency as heart rate. The change of conductivity caused by the CBFP is more significant than that of other intracranial pathophysiological activities. The CBFP causes periodic changes in the overall conductivity of the brain. Therefore, the information about CBFP can be theoretically reflected by MIPS signal.

### Measurement system

**Figure 2:**
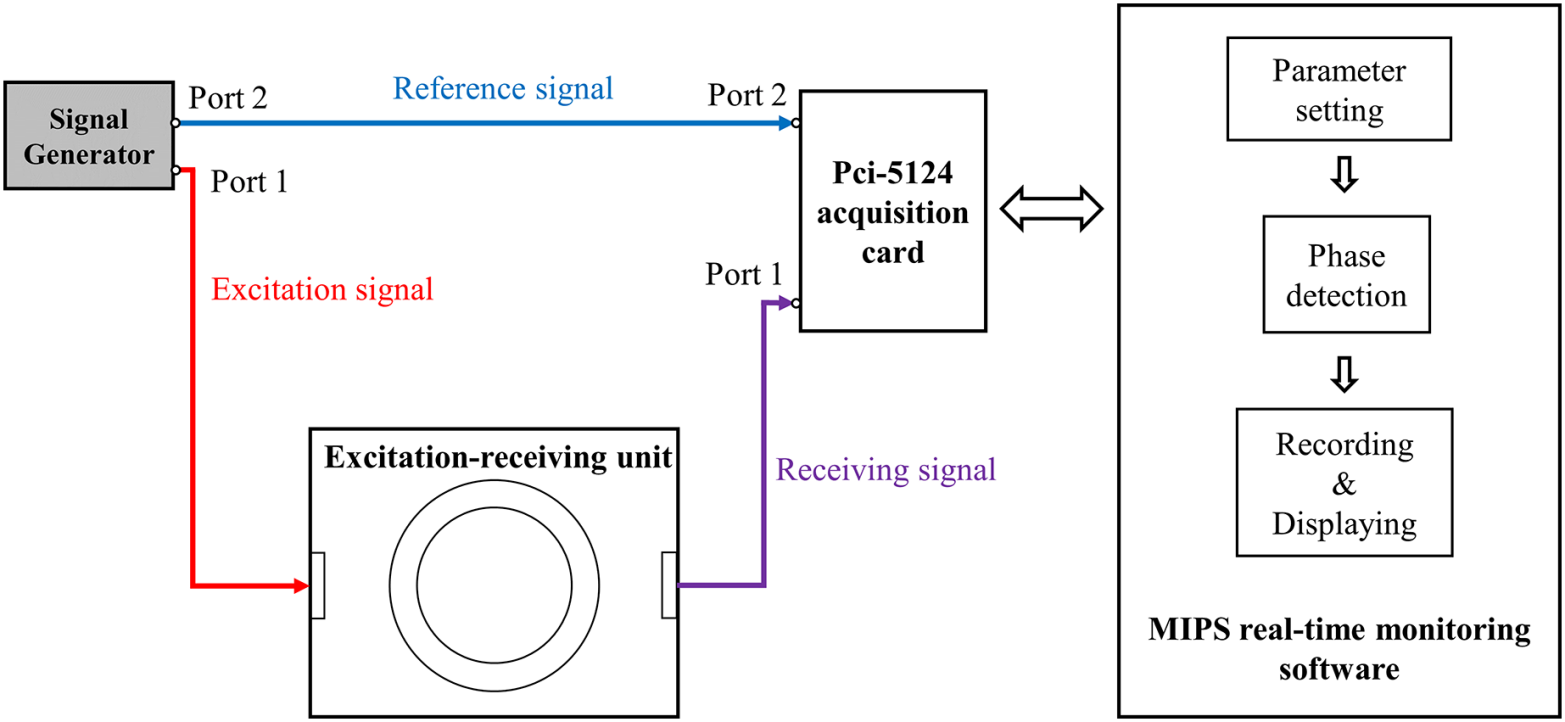
The measurement system for CBFP.

As shown in Fig. 2, the measurement system includes: a signal source (AFG3252; Tektronix, Beaverton, OR, USA), an excitation-receiving unit, a PCI-5124 acquisition card (National Instruments, Austin, TX, USA), and a MIPS real-time monitoring software.

Two spiral coils were used to form the excitation-receiving unit, where the inner ring was excitation coil and the outer ring was receiving coil. The coil parameters were: number of turns $N = 50$, inner radius ${r_{\rm inner}} = 50 \ {\rm mm}$, outer radius ${r_{\rm outer}} = 100 \ {\rm mm}$, wire diameter $l = 0.2\ {\rm mm}$, wire distance *d* = 0.2 mm. The maximum output power of the AFG3252 is 200 mW. It output two sinusoidal signals with the same frequency and phase ( $f = 10.7\ {\rm MHz}$, $\theta = 0$), where its port 1 with ${V_{pp}} = 5V$ was connected to excitation coil and port 2 with ${V_{pp}} = 1V$ was connected to port 2 of PCI-5124 as the reference signal. The receiving coil was connected to port 1 of PCI-5124. The software loaded on a PC (i7 2600k) was developed based on Labview platform and was used for parameter setting of PCI-5124, phase detection, data recording and displaying. The parameters of PCI-5124 were set as follows: data collection speed was 100 MHz, sampling point was 150,000, input impedance was 50 Ω. PCI-5124 collected the signals of port 1 and port 2 and transmitted them to PC. The PC calculated the MIPS data between the receiving signal and the reference signal by Fast Fourier Transform (FFT). Under these conditions, the sampling rate of MIPS data was 40 Hz.

**Figure 3:**
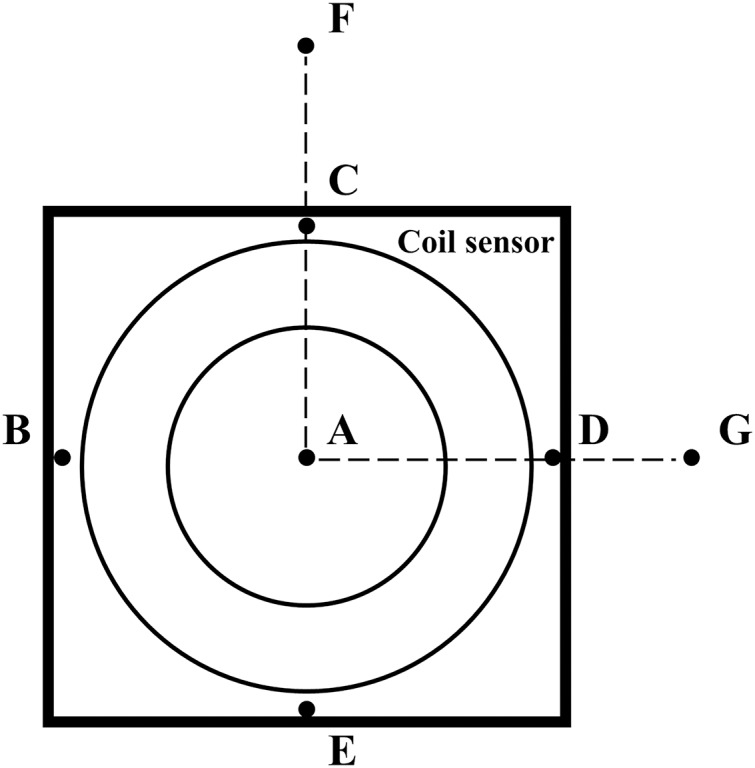
Measurement location of electromagnetic field strength.

Because this study involved human trials. The electromagnetic field strength of the MIPS detection system was tested by the magnetic field probe (HF3061, 300 kHz–30 MHz) and electric field probe (EF0391, 100 kHz–3GHz) of the electromagnetic field measuring instrument (NARDA NBM-550). As shown in Fig. 3, a total of seven points were measured. Among them, ABCDE are the points on the coil sensor, F is about 50 cm away from the vertical direction of the coil sensor center, and G is about 70 cm away from point A on the same plane as the coil.

### Construction of physical MCA model

A simplified MCA model was established to simulate cardiogenic CBFP. Practically, the pulsation of large vessels is more obvious. Elting et al. (2020) also pointed out that non-invasive microvascular-based cerebral autoregulation (CA) estimation and macrovascular-based CA estimation are similar. MCA has left and right sides and can be divided into 5 segments (M1–M5). Among these segments, M1 (horizontal segments) vessels are relatively larger and horizontal compared to other branches. The two main branches that are most prone to occlusion and thrombosis are from M1. When occlusion or embolism occurs at those branches, it will affect the cerebral blood flow velocity (CBFV) of M1 segment.

Based on the above anatomy, a silicone tube with a diameter similar to that of the MCA (inner diameter *D*_inner_ = 2.6 mm, outer diameter *D*_outer_ = 6 mm) for simulation was used in this study. The silicone tube was first drawn from the beaker and passed through the feeding pump (ZNB-XY1; KellyMed, Beijing, China); the total length of the feeding pump gear is *L* = 50 mm. Then, the silicone tube passed through the simulated cranial cavity from bottom to top and was fixed at the M1 segment position with a foam block. Finally, it was led out from the center of the forehead and back to the beaker. The end of the silicone tube was tied tightly, leaving only several pinprick holes. The beaker was filled with 0.9% saline solution ( $\sigma = 1.54\ {\rm S/m}$) to simulate blood. The feeding pump could regulate the flow rate ${v_{\rm flow}}$. Assume that the feeding pump squeezes $N$ times within 1 h. Denote the pulsation frequency (squeezing frequency) as ${f_p}$. The pumped volume within 1 h was $V$, then:

(5) $$N = {f_p}*3600$$

(6) $$V = \pi *{\left( {{D_{\rm inner}}/2} \right)^2}*L*N$$

### Physical experiments

**Figure 4:**
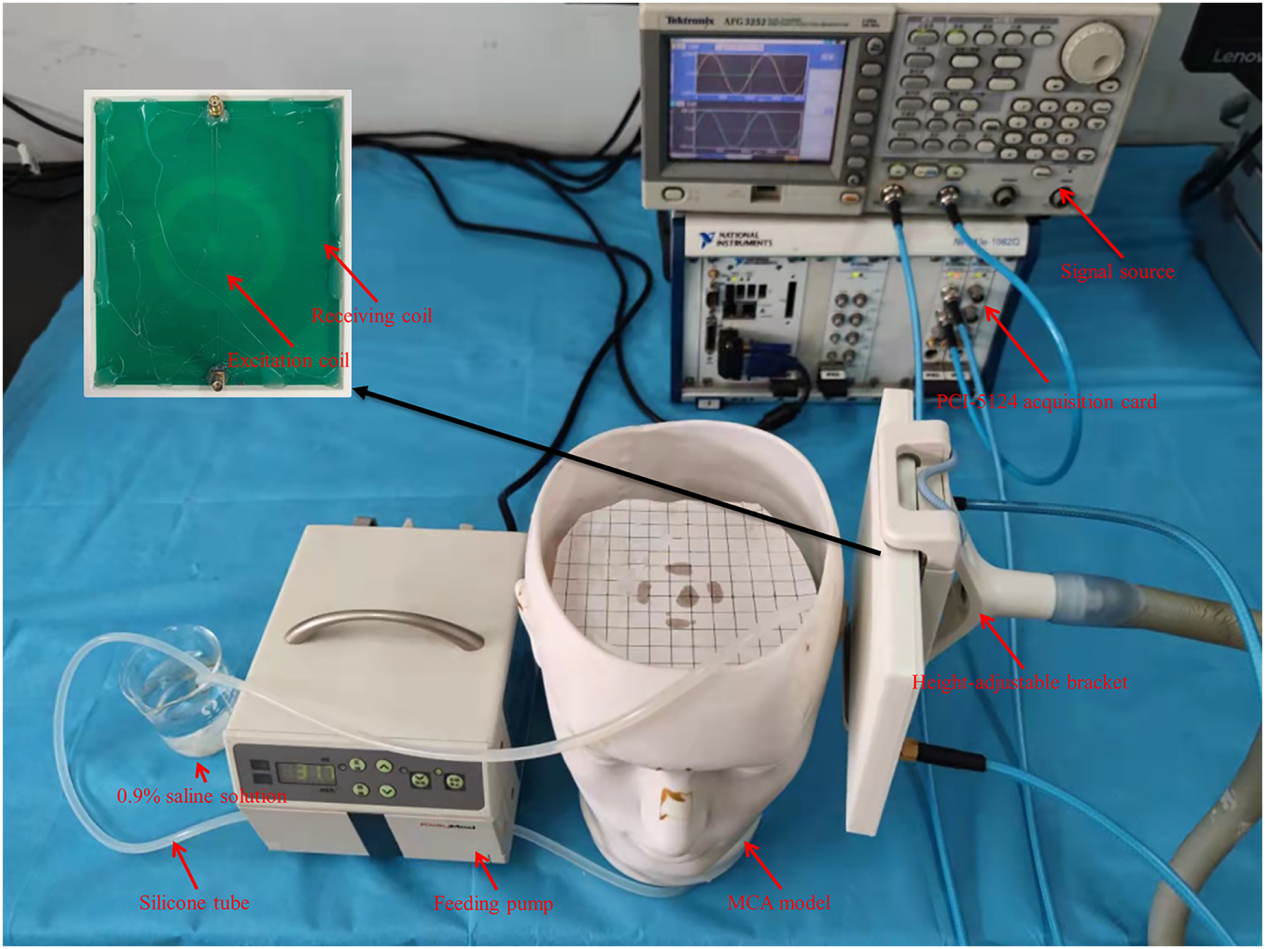
Experiments setup based on self-made physical MCA model.

The setup of the physical experiment is shown in Fig. 4. Firstly, the flow rate of peristaltic pump (*v*_flow_) was fixed at 1,000 ml/h, and the spatial resolution of MIPS detection was measured. The test range was 10 mm to 120 mm away from the coil, and the step was 10 mm.

The silicone tube was driven to produce different degrees of deformation by changing the *v*_flow_. The degree of deformation increased with the *v*_flow_, causing an increase in the diameter of the silicone tube and the volume of simulated CBF inside it. When the flow rate of the peristaltic pump ( ${v_{\rm flow}}$) is constant, the silicone tube forms a much smaller range of vibration than deformation. The process produces a dynamic change of volume similar to cardiogenic CBFP. And the beating frequency increased with *v*_flow_. Therefore, the *v*_flow_ was tuned to simulate different blood volume supply and CBFP level. The tune range of the *v*_flow_ was set to [0, 2,000] ml/h, and the step was 200 ml/h. In total there were 11 groups, which were sequentially marked as 0 ml/h, 200 ml/h, 400 ml/h,…., 2,000 ml/h. The experimental steps of different CBF supply levels are as follows. First, the measurement system warmed up for 0.5 h after power-on. Then, the *v*_flow_ was tuned sequentially (in 0 ml/h group, manually control the feeding pump to work until the silicone tube is filled with saline solution, and then stop). After setting parameters and waiting 30 s until the simulated CBF supply was stable, MIPS was measured for 30 s.

### MIPS and ECG synchronously monitoring trials

To further investigate the feasibility of monitoring CBFP based on the MIPS system, four healthy volunteers (male, aging from 20 to 30 years old) were enrolled for MIPS and ECG synchronously monitoring trials. The work was performed in accordance with the Declaration of Helsinki and was approved by the Ethics Committee of the First Affiliated Hospital of Third Military Medical University (Southwest, Hospital, Chongqing, China). For all research involving human subjects, informed consent to participate in the study had be obtained from participants. Protocol number of the approval: AF/12/058.0. Registered 20 December 2020.

**Figure 5:**
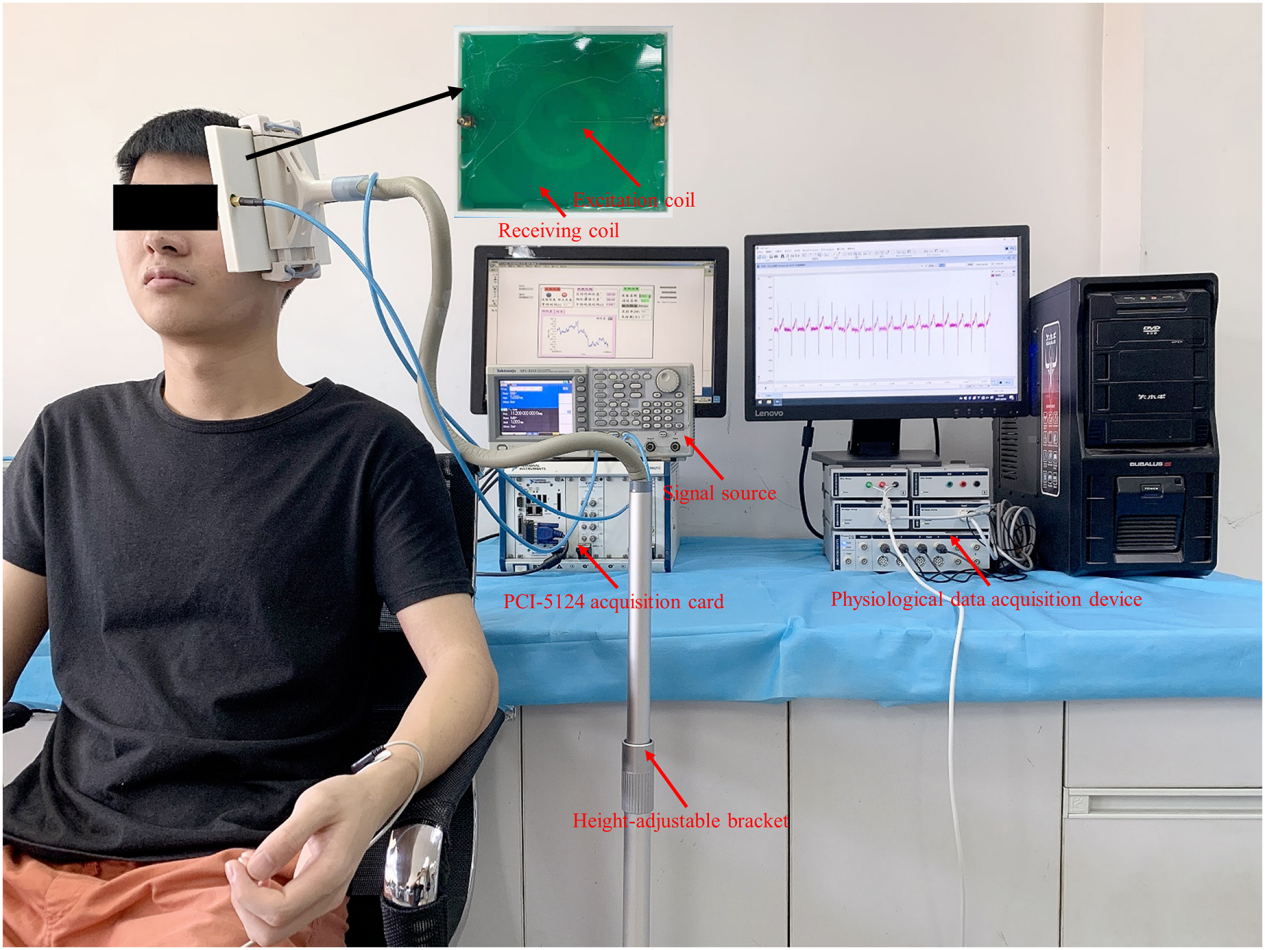
MIPS and ECG synchronously monitoring trial on healthy volunteers.

The excitation-receiving unit was fixed with a height-adjustable bracket, which ensured optimal detection position for subjects with different heights. As shown in Fig. 5, the subject remained sitting comfortably with the excitation-receiving unit placed on the left side of the head. When the subject was stabilized, MIPS data were recorded for 30-s. ECG signals were collected as a reference by the physiological data acquisition device (PowerLab 8/35; ADInstruments, Sydney, Australia). The ECG electrodes (MLA8203; ADInstruments, Sydney, Australia) were placed on the subject’s body in a four-lead manner. The initial sampling rate of the ECG signal was 1,000 Hz. After the experiment, it was resampled to 40 Hz and analyzed with the MIPS signal synchronously.

### Signal processing and analysis

**Figure 6:**
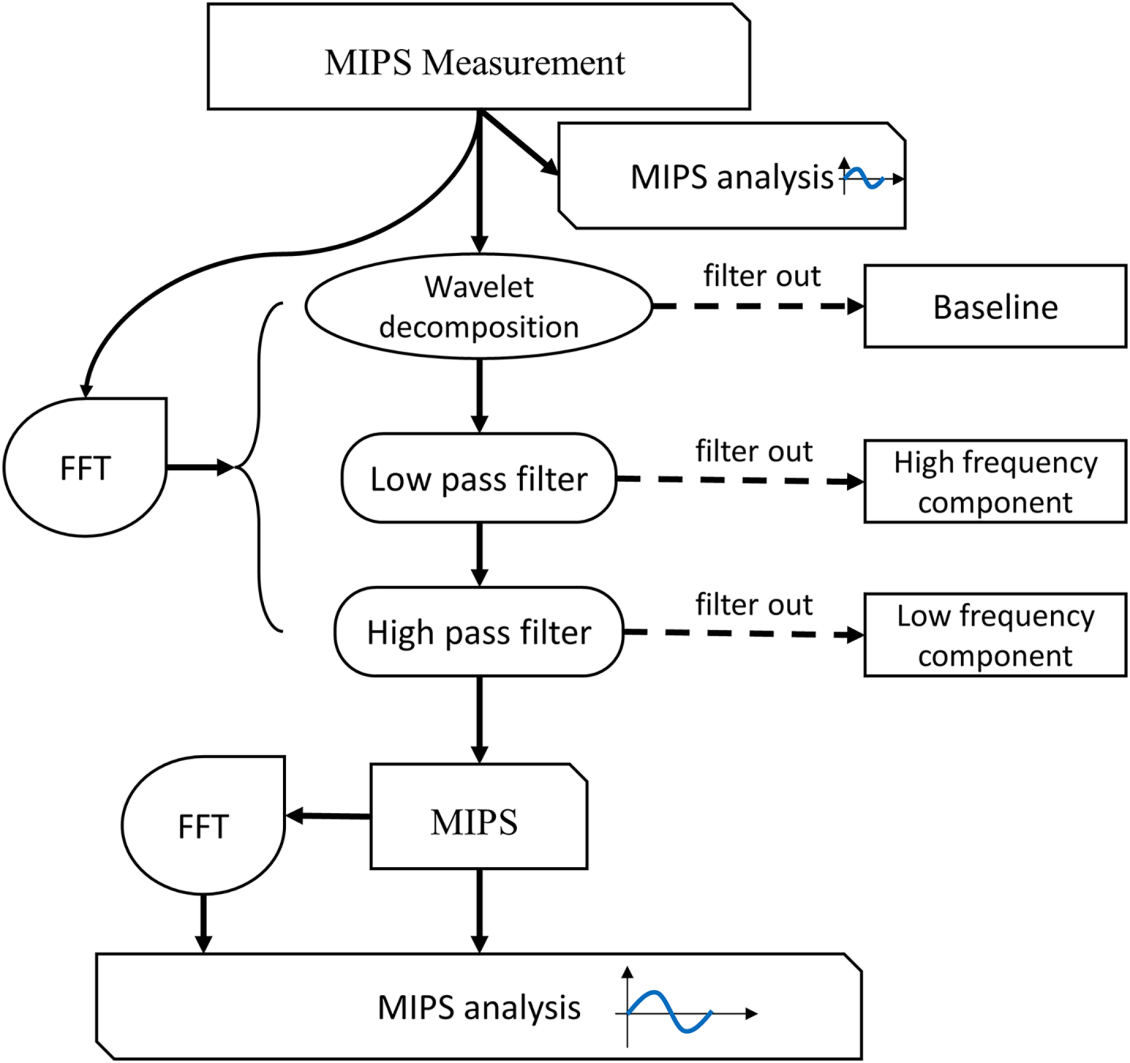
Flow chart of signal processing and analysis.

The flowchart of MIPS signal processing is shown in Fig. 6. Firstly, we observed the characteristics of the MIPS signal in time-domain and frequency-domain. Then wavelet transform was selected to filter out baseline drift. Finally, the CBFP component was extracted through high-pass and low-pass filtering. The processing in this study was performed by MATLAB R2015a (MathWorks Inc., Natick, MA, USA).

For physical experiment, first, we observed the baseline levels of MIPS signal at different deformation of the silicone tube, and investigated the feasibility of distinguishing different blood volume supply by MIPS. Then, the wavelet decomposition was used to filer the baseline drifts. The Butterworth high and low pass filters were used to extract CBFP components in MIPS signals. The selection of cutoff frequency was referenced by the vibration frequency of the silicone tube. The qualitative relationships between MIPS and different levels of cardiogenic CBFP were analyzed by comparing the amplitudes in time domain and the features in frequency domain of MIPS signals at different frequencies of vibration. The similar signal processing was performed in the MIPS and ECG synchronously monitoring trials. The cutoff frequency of the high and low pass filters was related to the heart beat (1.2–1.3 Hz) of the volunteers. After processing, the MIPS and the resampled ECG data were analyzed simultaneously.

### Statistical analysis

The MIPS data in time and frequency domain were statistically analyzed to investigate the effectiveness in detecting different levels of CBFP. Before processing, we calculated the mean ± standard deviation of MIPS monitoring data at all flow rates from [0, 2,000] ml/h, which represented the baseline. Linear fitting analysis was carried out with mean MIPS as dependent variable and flow rate as independent variable. The pulsation signals and mean values of MIPS from [200, 2,000] ml/h were also analyzed by linear fitting. The significance level was set at *p* < 0.05. Statistical analyses were performed by Origin 9.1 (Origin Lab, Northampton, MA, USA).

A Pearson cross-correlation analysis (α = 0.05) on the MIPS and ECG data of four healthy volunteers was performed to quantify the delay time between heart beat and CBFP. The synchronous monitoring results with a time length of 4 s were selected for analysis. The MIPS signal was fixed, the ECG signal in a time window of 3 s was moved to the zero time direction with the minimum sampling interval, and the Pearson correlation and *p* value were calculated by IBM SPSS Statistics 22 (IBM, New York, NY, USA). According to the physiological mechanism of cardiogenic CBFP, ECG is theoretically positively correlated with MIPS. Therefore, the delay time is determined when the Pearson correlation is maximum and the *p* value is less than the significance level (α = 0.05).

## Results

The measurement results show that the electric field strength at C is the strongest (9.47 V/m), which is basically the same level of magnitude as that of ABDE. The strength decays rapidly with increasing distance from the central location. The magnetic field strength at B is the strongest at 0.0252 A/m, which is almost at the same level as ACDE. The magnetic field strength gradually decays with increasing distance. The excitation frequency in this study is 10.7 MHz, and the theoretical electric limit and magnetic field strength limit are 45 V/m and 0.12 A/m respectively. Therefore, the electric and magnetic field strengths in this work are far below the corresponding limits, which are in line with safety standards. In addition, the maximum output power of the signal source is 200 mW and the frequency of the excitation signal is much lower than the microwave frequency band. There will be no obvious thermal effect under the experimental conditions in this study.

The experimental results of spatial resolution are shown in Table 1. MIPS continues to decrease as the distance increases from 10 mm to 90 mm. When the distance exceeds 90 mm, MIPS has no obvious upward or downward trend. This result shows that the maximum detection range of the coil sensor is 90 mm. Besides, there is no linearity between the distance and the MIPS variation in the detection range. When the distance increases by 10 mm, the variation of MIPS ranges from 0.07° to 0.97°.

**Table 1:** MIPS test results from distance 10 mm to 120 mm at 1,000 ml/h.

Distance (mm)	Phase differences (°)
10	20.56
20	19.59
30	19.22
40	18.94
50	18.87
60	18.76
70	18.68
80	18.60
90	18.55
100	18.63
110	18.60
120	18.59

**Figure 7:**
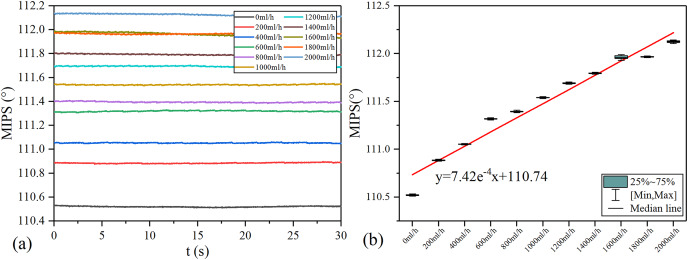
MIPS trend in each group. (A) MIPS signal within 30 s. (B) MIPS trend as a function of flow rates and fitting line.

MIPS results in each group of *v*_flow_ are shown in the Fig. 7. The volume of simulated CBF inside the silicone tube increases with *v*_flow_. As shown in Fig. 7A, the baselines (mean value) of MIPS in each group are different, accompanied by different degrees of drift and clutter interference. Fig. 7B is the box plot of MIPS data within 30 s for each *v*_flow_ group. It shows that MIPS has an approximately linear upward trend with the increase of *v*_flow_. The relationship between MIPS baseline values and the *v*_flow_ can be obtained by linear fitting where ${\rm MIPS} = 7.42{{\rm e}^{ - 4}}\ {v_{\rm flow}} + 110.74$ ( ${R^2} = 0.956$). Significance test results show that the $F$ is 217.266, corresponding to $P \lt 0.05$ (Table 2). It indicates that there are significant differences in MIPS detection results among different volumes of simulated CBF. According to Eq. (4), MIPS is correlated to the geometric parameter $P$. This trend is consistent with the theoretical inference. These results illustrate that MIPS is able to distinguish different blood volume supply levels. Moreover, an increase in *v*_flow_ causes the diameter expansion of the silicone tube. At the same time, the silicone tube tension decreases as the diameter expands. So the relationship between *v*_flow_ and the liquid pressure in this model is not completely linear. Furthermore, the silicone tube vibrates at a certain frequency on the basis of deformation when the flow rate of the peristaltic pump unchanged, which causes the volume of the internal solution to change periodically. Because the pressure drive of the peristaltic pump is relatively weak, the vibration of the silicone tube is significantly weakened or even disappeared after it is fixed. Unfixed silicone tube produces random and slight shaking during the vibration process, causing the measurement result to drift up or down.

**Table 2:** Anova to determine relationship between MIPS and simulated volume of CBF.

Model		Sum of squares	df	Mean squares	F	Sig.
1	Regression	2.421	1	2.421	217.266	1.31E−07
	Residual	0.100	9	0.011		
	Total	2.521	10			

**Figure 8:**
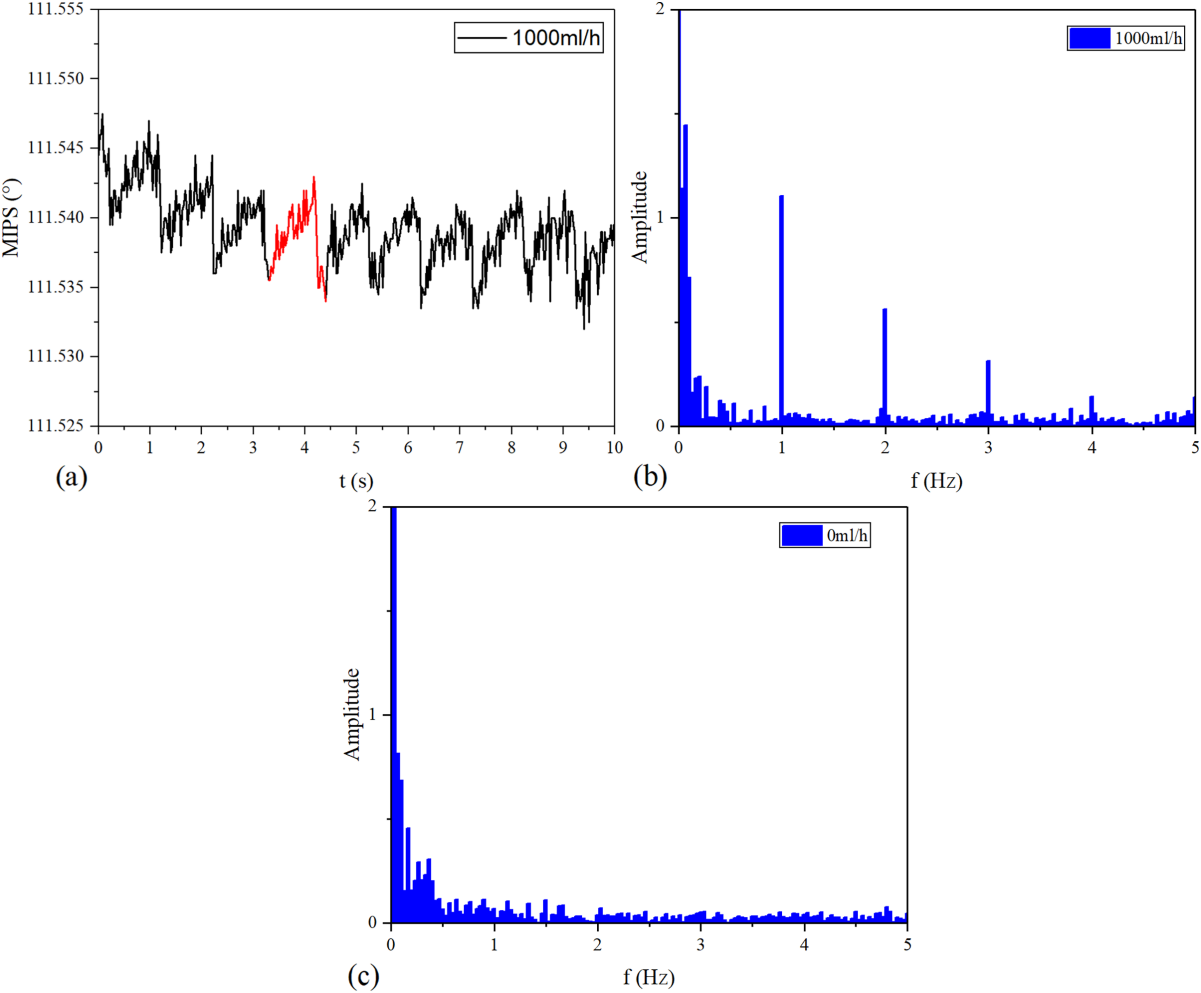
Time domain and frequency domain signal of MIPS. (A) MIPS at the flow rate of 1,000 ml/h. (B) Spectrum of 1,000 ml/h group. (C) Spectrum of 0 ml/h.

The MIPS signals in time and frequency domains are shown in Fig. 8. Continuous vibration of the silicone tube makes the volume of simulated CBF change periodically. Figure 8A is the MIPS in a short period of 1,000 ml/h group. Obviously, there is a pulsation component in the MIPS signal with the periodic change in volume of simulated CBF. Figures 8B and 8C draw out the frequency spectrum of MIPS in 1,000 ml/h group and 0 ml/h group. In Fig. 8B, there is an obvious signal near 1 Hz. By comparison, in 0 ml/h group (the feeding pump didn’t work), there was no obvious frequency component in the spectrogram. When ${f_p} = 1\ {\rm Hz}$, it can be calculated from Eqs. (5) and (6) that $V \approx 956\ {\rm{ml}}$. Considering that the accuracy of ZNB-XY1 is $\pm 10\%$, it can be concluded that the result is correct. This shows that the MIPS signal contains the pulsation component of cerebral blood volume. Therefore, the signal can be transformed by FFT to observe the frequency component of this pulsation and filter out interference.

The frequency of periodic simulated CBF volume change increases with the vibration speed of the silicone tube. And the vibration speed of the silicone tube increases with the *v*_flow_.

**Figure 9:**
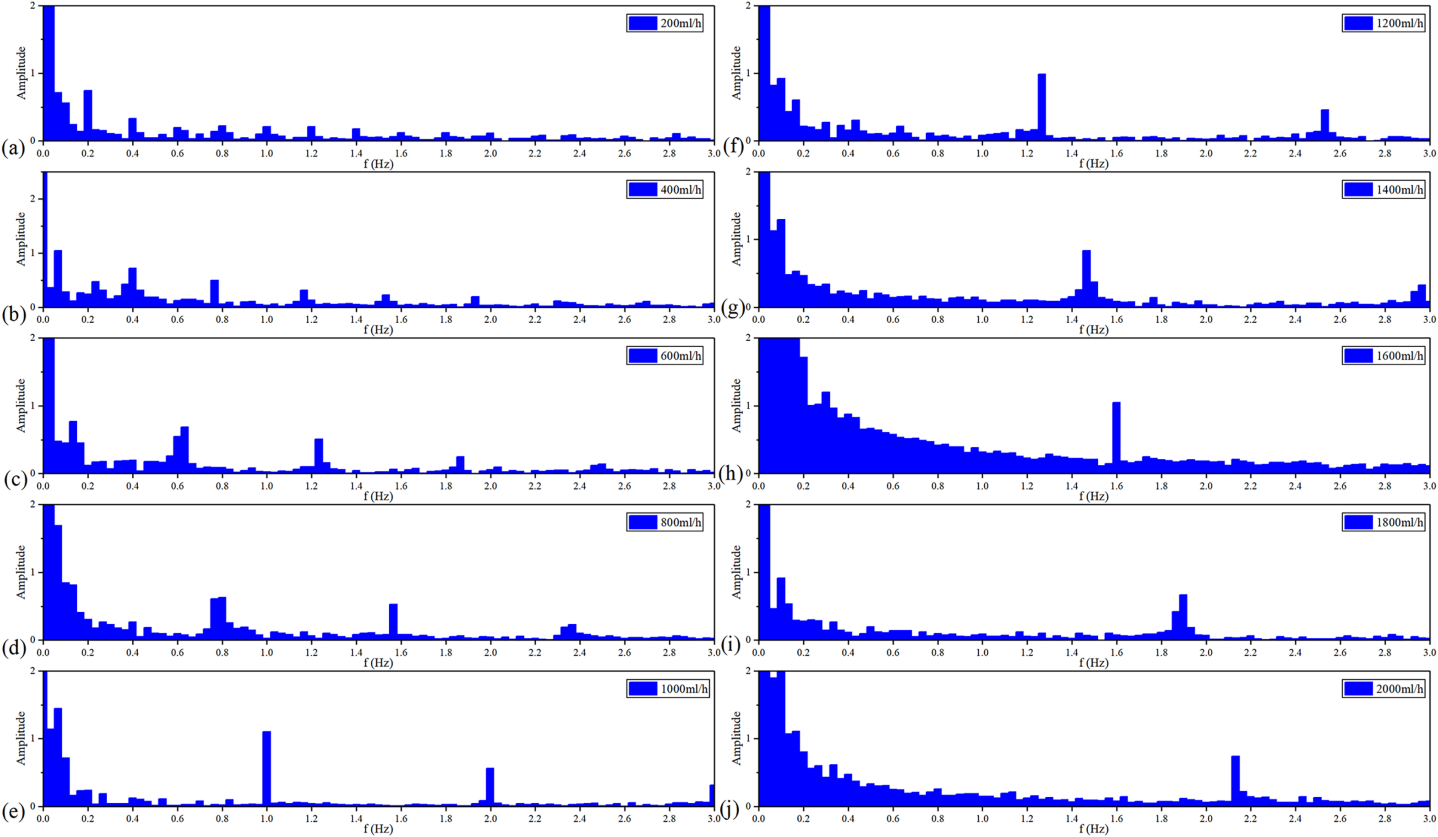
Spectroscopy analysis results of MIPS signal in each flow rate.

Figure 9 shows the spectroscopy analysis results of MIPS in each group of the *v*_flow_. With the acceleration of the *v*_flow_, each group has a signal component from 0.2–2 Hz in sequence with a step of 0.2 Hz. Each group meets the ratio of ${v_{\rm flow}}/{f_p} = 1,\!000$. It proves that MIPS method can detect the simulated CBFP and obtain the pulsation speed change.

**Figure 10:**
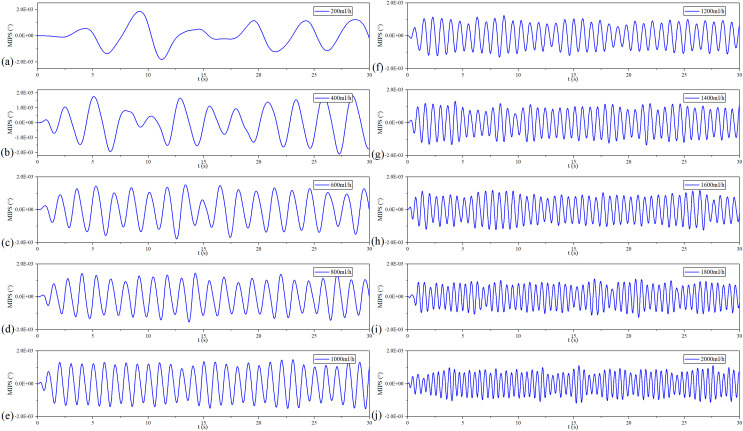
Filtered MIPS signal in each flow rate.

**Figure 11:**
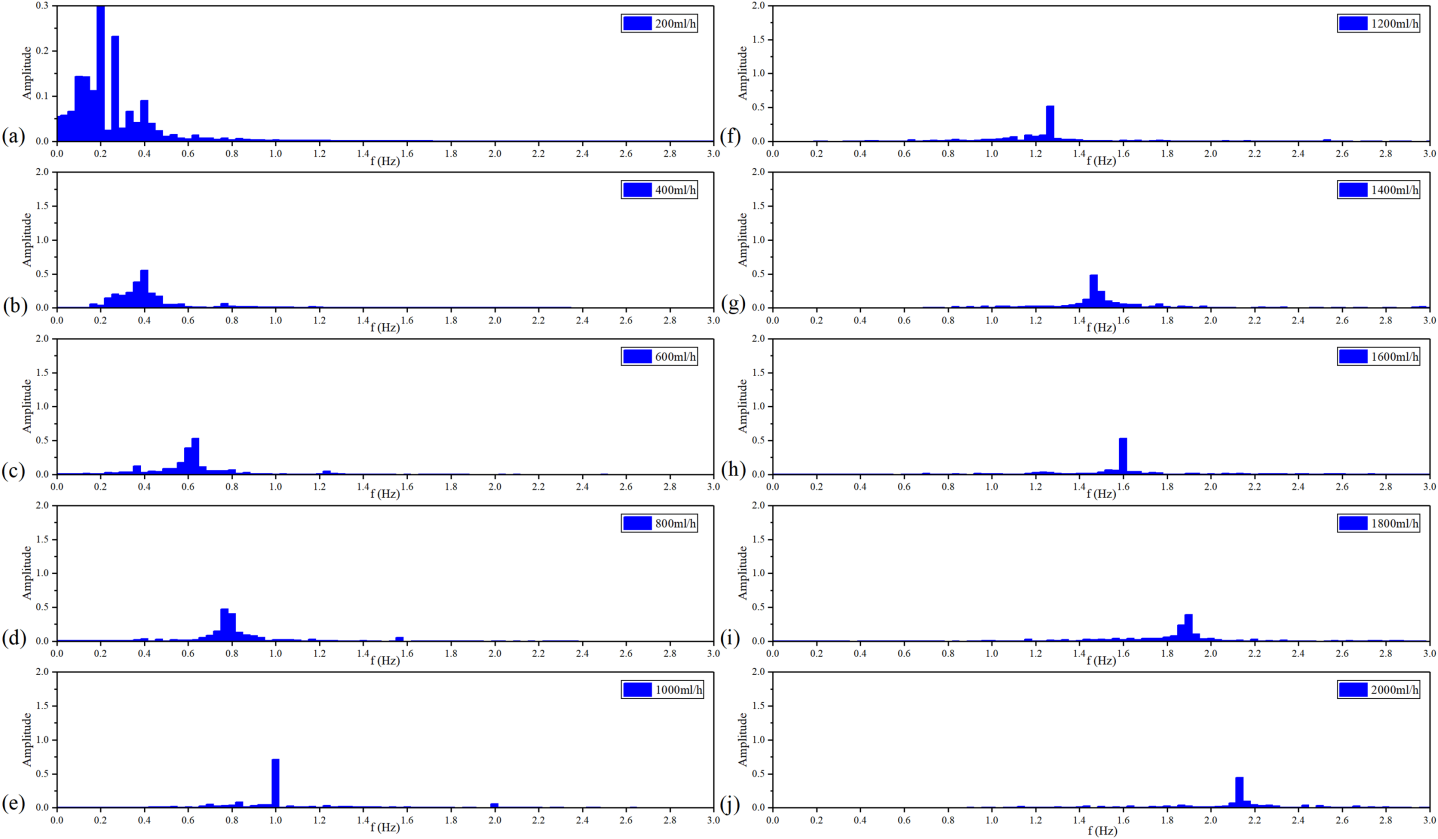
Spectroscopy analysis results of filtered MIPS signal in each flow rate.

Figure 10 shows the filtered MIPS signals. As a result, the saline solution in the silicone tube is pulsating in a sinusoidal rhythm. Further, the faster the flow rate, the higher the frequency of filtered MIPS signals. Figure 11 shows the spectroscopy analysis results of the filtered MIPS signal in each group of the *v*_flow_. With the increase of vibration speed of the silicone tube, the features of the MIPS signals in frequency domain have corresponding shifts. But variations of their amplitudes are nonlinear. In the initial stage of the increasing *v*_flow_, the silicone tube has a better elasticity and a larger vibration range, so that the volume of simulated CBF has a relatively large periodic change. With the further increase of the *v*_flow_, both the vibration range of the silicone tube and the periodic pulsation amplitude of simulated CBF have nonlinear changes. MIPS detects the volume change of simulated CBF in the silicone tube. Therefore, the amplitudes of processed MIPS signals vary nonlinearly with the increasing *v*_flow_. The frequency drifts and the baselines of MIPS from [200, 2,000] ml/h can be fitted linearly and the ${R^2}$ is 0.970. Significance test results show that the $F$ is 262.065, corresponding to $P \lt 0.05$ (Table 3). It indicates that there is a significant difference in MIPS signal at various frequencies of CBFP. These results illustrate that the speed and amplitude of CBFP can be reflected by features of MIPS in time and frequency domain.

**Table 3:** Anova to determine relationship between frequency drifts and the baselines of MIPS.

Model		Sum of squares	df	Mean squares	F	Sig.
1	Regression	3.606	1	3.606	262.065	2.13E−07
	Residual	0.110	8	0.014		
	Total	3.716	9			

**Figure 12:**
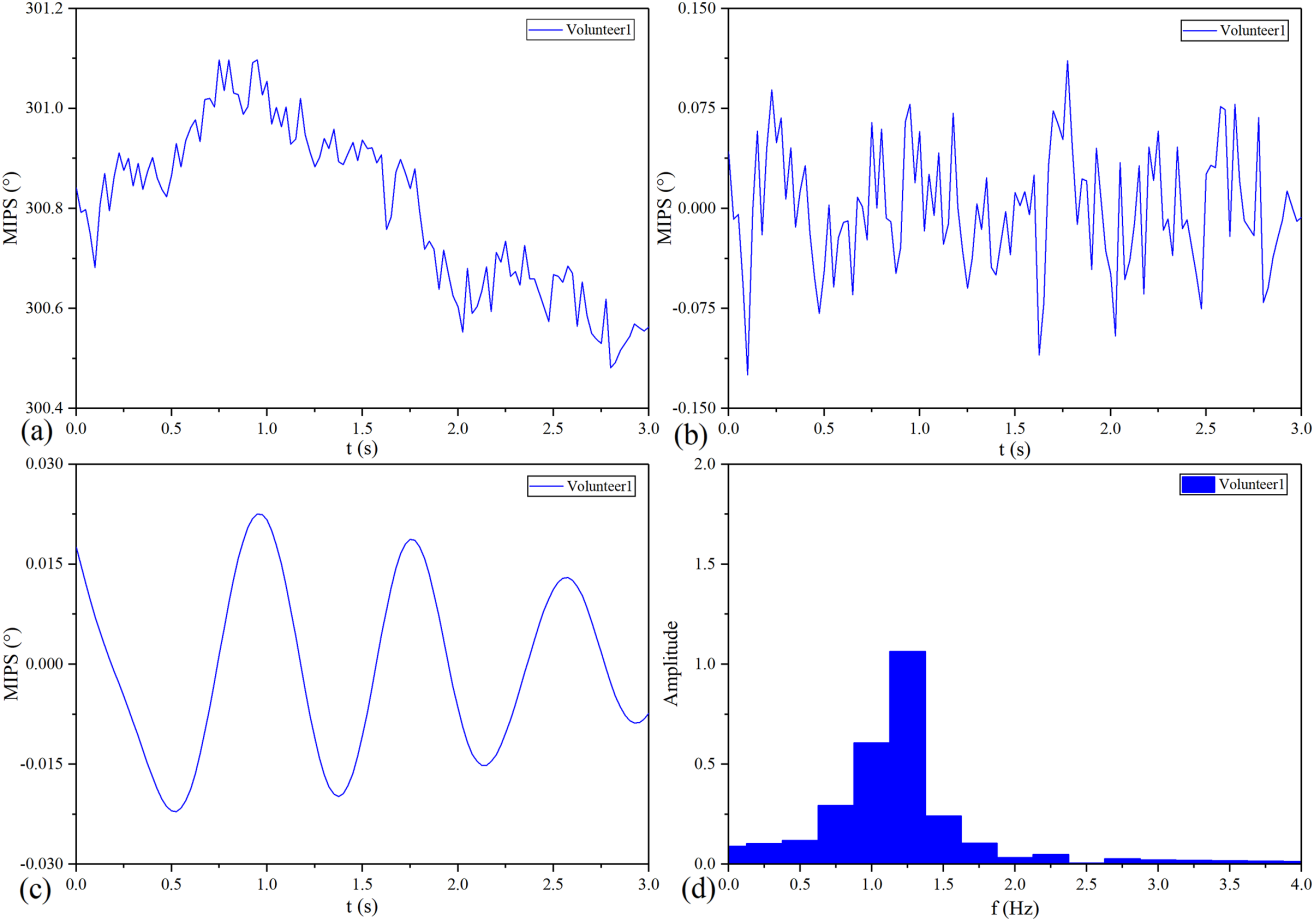
MIPS signal before and after processing. (A) Original MIPS signal of No.1 healthy volunteer. (B) MIPS signal of No.1 healthy volunteer after removing baseline drift. (C) MIPS signal of No.1 healthy volunteer after high-pass and low-pass filtering (D) MIPS signal of No.1 healthy volunteer in frequency domain.

The MIPS signal of No.1 healthy volunteer before and after processing is shown in Fig. 12. Similar to the pulse wave, the original MIPS shown in Fig. 12A has periodic changes but contains some glitches and baseline drifts. These baseline drifts mainly come from body movement, respiration and temperature difference between the coil sensor and the scalp. As shown in Figs. 12B and 12C, we removed the baseline drifts and obtained the MIPS signals by wavelet decomposition and Butterworth high- and low-pass filtering. Figure 12D shows that the main component of processed MIPS signal is concentrated around 1.2 Hz, which is consistent with the frequency of heartbeat.

**Figure 13:**
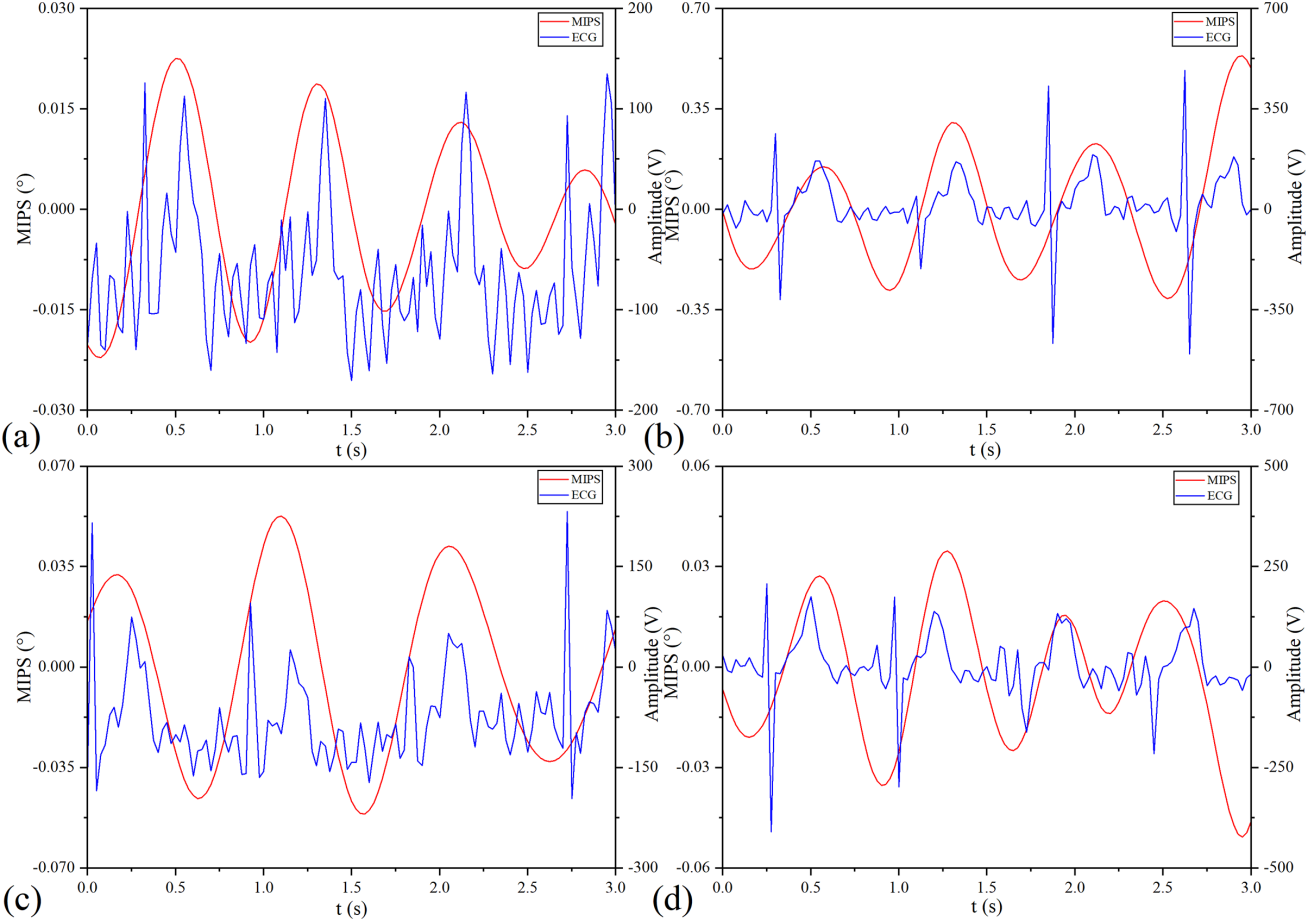
MIPS and ECG synchronously monitoring outcomes. (A–D) represent the monitoring outcomes of four subjects respectively.

MIPS and ECG synchronously monitoring results of the four volunteers are shown in Fig. 13. The four groups of MIPS signals all have periodic change trends with obvious peaks and valleys, which are consistent with the results of physical experiments. More importantly, the frequency of the MIPS is close to that of the ECG signal and there is a certain time delay between them. This is consistent with the mechanism that CBFP and heart beat have the same frequency but different phases. Table 4 shows the results of Pearson cross-correlation analysis in healthy volunteer trials. The average delay time of the four healthy volunteers is 0.375 s, the *p* values are all less than 0.05 (α = 0.05), and the Pearson correlation values are all less than 0.8. There is a weak cross-correlation relationship between them. These results strengthen that the MIPS signal is able to reflect the cardiogenic CBFP.

**Table 4:** Pearson cross-correlation analysis in healthy volunteer trials.

Subject	Delay time (ms)	Pearson correlation	Sig.
Volunteer 1	0.425	0.449	2.445 × 10^−7^
Volunteer 2	0.325	0.316	4.232 × 10^−4^
Volunteer 3	0.175	0.349	8.982 × 10^−5^
Volunteer 4	0.575	0.329	2.248 × 10^−4^

## Discussion

The loss of CBF is closely related to the poor prognosis of cerebrovascular diseases. Biesbroek et al. (2013) has investigated the sensitivity and specificity of (CTP) for the diagnosis of ischemic stroke based on a systematic review and meta-analysis. The results of the study in 1,107 patients have shown a sensitivity of 80% and a specificity of 95%, indicating that CTP has high sensitivity and specificity. There is also evidence that timely CTP imaging of patients is helpful for reperfusion therapy and prognosis. Ritarwan et al. (2020) have conducted a comparative study of TCD and Hematological Parameters in patients with ischemic stroke and have found that in 90 patients, the middle cerebral artery pulsation index is positively correlated with the NIHSS score. The real-time assessment of CBFP is helpful for clinicians to intervene loss of perfusion in time, and is of great significance to avoid the occurrence of secondary pathophysiological reactions in post stroke.

MIPS can theoretically realize non-invasive continuous monitoring of cardiogenic CBFP based on changes in the overall conductivity of the brain. The key of the MIPS detection system is to meet the requirement of phase detection accuracy. Reported work by Watson, Williams & Griffiths (2003) has illustrated that the accuracy needs to reach 0.01° when the MIPS of biological tissue is measured using the excitation frequency of 10 MHz. The previous researches of our research group have shown that the phase detection accuracy of the MPS detection system based on the NI-PCI data acquisition card is able to reach 0.01° and the detectable minimum volume of intracranial blood is 0.5 ml (Jin et al., 2014a, 2014b). Under such phase detection accuracy and intracranial blood detection sensitivity, one phase difference measurement time in this study is 0.025 s (40 Hz), thus, the proposed method has a very high time resolution compared with imaging methods such as CT and MRI.

The MIPS measurement system established in this work can assess the different levels of CBFP in real-time. After ischemic stroke, the diameter of some large blood vessels will change under the stimulation of sympathetic nerves and the infusion of vasoactive drugs (Kim et al., 2018). Clinical evidence shows that the diameter of cerebral arteries is related to CBFP (Zeiler et al., 2020). However, the TCD method is difficult to reflect CBFP accurately under this situation (Moerman & Hert, 2019). For this, we propose a comprehensive continuous assessment method of CBFP based on MIPS. In the physical experiment, the silicone tube is deformed to different degrees by setting the flow rate of the peristaltic pump (*v*_flow_). At this time, the diameter expansion of the silicone tube caused by the deformation will cause an increase in the volume of simulated CBF. In addition, the silicone tube will vibrate on the basis of deformation. The faster the *v*_flow_, the higher the vibration frequency. This will cause periodic changes in simulated CBF volume, which is consistent with the mechanism of cardiogenic CBFP. And its change amplitude is much smaller than that induced by deformation. The baseline level of MIPS increases with the CBF volume caused by the deformation. After Fourier transform of MIPS signal in each group of *v*_flow_, in addition to the fundamental wave component with the same frequency as the vibration frequency of the silicone tube, there are a series of harmonic components higher than it. These harmonic components are also related to the volume of simulated CBF and should not be ignored. However, the CBFP signal is relatively weak. It is easily disturbed by baseline drift and power frequency noise. And the difference in CBFP is mainly reflected in the amplitude of MIPS. In order to qualitatively analyze the feasibility of MIPS to detect CBFP, we assume that the volume change caused by the vibration of the silicone tube is a sine wave, and extract the fundamental component of the MIPS signal with the highest amplitude. It increases with the vibration frequency of the silicone tube. These results indicate that continuous monitoring of MIPS signals can distinguish different CBF volume levels and reflect cardiogenic CBFP.

MIPS is able to realize real-time monitoring of CBFP in healthy volunteers. Similar to the artery pulse wave, the CBFP is a periodic signal produced by blood pumping from the heart to brain and its frequency is close to the heartbeat. Since the conductivity and volume change of CBF is higher than other brain tissues, we can theoretically obtain CBFP in each cardiac cycle through MIPS. In the healthy volunteer trials, the original MIPS signal contains a main peak and a double pulse wave in each cycle and its frequency is close to the ECG. Same as the physical experiment, the fundamental component close to the ECG frequency in the MIPS signal has been extracted. As an electrophysiological signal, there is no direct correlation between ECG and mechanical beat of cardiac. But the intervals among the QRS peaks can indirectly reflect the heartbeat. The frequencies of the processed MIPS signals in the four healthy volunteers are consistent with their ECG and the amplitudes in the time domain are basically the same level. Combined with the results of physical experiments, it shows that the MIPS signal contains components of CBFP. We can construct the attenuation index of CBFP based on the amplitude of MIPS to evaluate the level of that in different pathophysiological states. But this is only a preliminary study, and relying solely on one parameter may result in poor specificity. In the future, we will investigate methods to reliably detect CBFP of left and right hemispheres to improve accuracy and sensitivity. Then, the MIPS signal containing the information about CBFP in systolic and diastolic periods will be extracted by wavelet threshold processing, and abundant features will be obtained by the method of similar pulse wave feature point recognition.

The assessment of cardiac and cerebral delay level plays a role in the prevention of acute cerebrovascular disease. It will also be interesting to distinguish this delay time with different heights. There is a certain time delay between ECG and MIPS in this study, corresponding to the delay between cardiovascular and cerebral rhythms (Bernardi et al., 2009). The delay between cardiovascular and cerebral rhythms will change with the decline of body function (Tsivgoulis et al., 2019). We deem that MIPS can capture this information. Combined with Pearson cross-correlation analysis, we preliminarily quantified the delay time according to the simultaneous monitoring results of ECG and MIPS. The mean delay time of the four healthy volunteers is 0.375 ms. The size of this delay is related to the height, age, physical condition and other factors of subjects (Lin et al., 2016). The volunteers in this work are aging from 20 to 30 years old without chronic cardiovascular and cerebrovascular diseases. Therefore, their mean delay time can be regarded as a feature to identify CBFP in different pathophysiological states. However, there is no direct pathophysiological relationship between ECG and CBFP, resulting in a weak cross-correlation relationship between MIPS and ECG signals. In the future, we will monitor heartbeat and CBFP synchronously based on MIPS technology to accurately quantify their delay time. In addition, a group of healthy volunteers with obvious height differences will be selected to study the relationship between delay time and height.

This work extracts the physiological parameters related to neurosurgical monitoring, and promote the development of stroke monitoring technology based on bioelectromagnetics. Previous studies have achieved excellent outcomes in the diagnosis and monitoring of cerebral hemorrhage, brain edema, ischemic stroke and so on (Gonzalez et al., 2013; Kandadai et al., 2014; Gonzalez et al., 2009; Pan et al., 2015). It is proved that some parameters (*e.g*. cerebral blood oxygen, intracranial pressure, CBFP) are closely related to the prognosis (Amki & Wegener, 2017; Chen et al., 2019; Khalil et al., 2017). The establishment of monitoring models for those parameters based on electromagnetic signals will make it more acceptable in clinical neurosurgery monitoring. For this, we’ve built an early warning model of intracranial hypertension after traumatic brain injury. Unfortunately, the detailed features of the ICP waveform have not been paid enough attention (Li et al., 2021). Based on non-ionizing radio frequency electromagnetic waves, Oziel et al. (2016) have obtained different responses of CBFP under the blood pressure changes. Their work has provided a basis for this study extracting CBFP information based on MIPS signals. CBFP at different levels can be distinguished more clearly through observation of its characteristics in time and frequency domains. In addition, the healthy volunteer trials have also provided a reference for the monitoring of stroke.

However, there are some limitations in this work: (i) Vibrations of different frequencies and amplitudes are generated in a single-mode by the physical model. Actually, the pulsation of cerebral arteries is based on the superposition of multiple vibration modes. (ii) The levels of CBFP are related to many factors, we only verified the feasibility of MIPS to detect volume changes induced by cerebral artery diameter expansion and pulsation. (iii) At present, ECG and MIPS signals are collected based on different instruments, leading to certain systematic errors. We will develop an integrated synchronous monitoring system of MIPS and ECG. (iv) Although the coil sensor in this study is not in contact with the human body, conductivity changes caused by skin hydration, ion and metabolite fluctuation, skin temperature, and sweat all reduce the MIPS detection accuracy. In addition, the change of the relative position between the coil sensor and the head produces some motion artifacts on the MIPS signal.

## Conclusions

In order to realize the non-invasive, continuous and comprehensive evaluation of CBFP, we propose a new solution based on MIPS which detects changes in the brain’s overall conductivity. The results of physical experiment through a self-made MCA model indicate that the MIPS can distinguish different CBF volume supply and detect CBFP at different levels. The periodic MIPS signals similar to pulse waves are obtained in healthy volunteer trials, and the frequency is close to the ECG. Furthermore, there is a certain time delay between ECG and MIPS, which is consistent with the mechanism of the delay between cardiovascular and cerebral rhythms. Overall, MIPS has the potential to become a non-invasive, continuous and comprehensive monitoring method of cardiogenic CBFP.

## Supplemental Information

10.7717/peerj.13002/supp-1 Supplemental Information 1 Data of the physical experiments.

10.7717/peerj.13002/supp-2 Supplemental Information 2 Data of MIPS and ECG synchronously monitoring trials.

## Additional Information and Declarations

### Data Availability

The following information was supplied regarding data availability: The raw measurements are available in the Supplemental Files.
